# Analytical Method for the Validation of Three Polyphenols as a Marker Compound for the Standardization of* Solidago virgaurea* subsp.* gigantea* Extracts and Antiadipogenesis of Harvesting Time and Location

**DOI:** 10.1155/2017/3047408

**Published:** 2017-07-12

**Authors:** Seung Hwan Hwang, Ju Hee Kim, Zhiqiang Wang, Jae-Yong Lee, Soon Sung Lim

**Affiliations:** ^1^Department of Food Science and Nutrition, Hallym University, 1 Hallymdeahak-gil, Chuncheon 24252, Republic of Korea; ^2^Institute of Natural Medicine, Hallym University, 1 Hallymdeahak-gil, Chuncheon 24252, Republic of Korea; ^3^Department of Biochemistry, School of Medicine, Hallym University, 1 Hallymdeahak-gil, Chuncheon 24252, Republic of Korea

## Abstract

Protocatechuic acid (PC), chlorogenic acid (CA), and kaempferol-3-*O*-rutinoside (K-*O*-R), isolated from the* Solidago virgaurea* subsp.* gigantea* (SV) extract, were quickly and efficiently separated using HPLC. Our chromatographic method was found to effectively separate PC, CA, and K-*O*-R at retention times of 5.36, 8.22, and 17.04 min, respectively. Linearity of PC, CA, and K-*O*-R was found to be in the range of 4.85–485.00, 47.5–1900.00, and 8.50–850.00 *μ*g/ml. Recoveries ranged between 101.32 and 103.30%, 95.82 and 100.25%, and 96.18 and 99.37%, for PC, CA, and K-*O*-R, respectively. The antiadipogenesis activity of SV extracts collected from five different months and from seven different regions was evaluated using an Oil Red O staining assay in 3T3-L1 cells. Extract from SV collected in April from the Ulleung Island produced over 106.89% inhibition of adipogenesis without cytotoxicity at 50 *μ*g/ml. This extract had a high amount of PC and K-*O*-R. The developed HPLC method was found to be fast, accurate, precise, and reproducible and could be applied to qualitative and quantitative analysis of three bioactive compounds in SV extracts. The SV extract collected in April from Ulleung Island can be used as a functional food ingredient preventing obesity.

## 1. Introduction

*Solidago virgaurea* subsp.* gigantea* (SV) is a perennial herb known as Goldenrod, which grows in Ulleung Island in Korea [1]. Whole parts of SV have been used traditionally as folk medicine in Korean as an anti-inflammatory for the treatment of various symptoms [2]. Various* Solidago* species have been used to treat indigestion and infections and have been shown to have antibacterial, antioxidant [3], and anti-inflammatory activities [4], as well as elevating bone metabolic activity [5]. The known chemical constituents of SV have been reported to include caffeoylquinic acid derivatives, triterpenoid saponins, quercetin derivatives, kaempferol derivatives, and anthocyanidins [6–8].

Protocatechuic acid (PC), chlorogenic acid (CA), and kaempferol-3-*O*-rutinoside (K-*O*-R) have been found as natural products of a variety of species, including* Hippophae rhamnoides* L. [9],* Xanthium strumarium* [10], and* Carthamus tinctorius* L. [11]. PC has been shown to activate the AMPK/mTOR/S6K pathway in cultured cells in vivo and improve glucose tolerance and insulin sensitivity in obese mice that were models of early stages of Type 2 diabetes [12]. Ong et al. (2013) and Ma et al. (2015) reported that CA improved glucose and lipid metabolism, via the activation of AMPK, and blocked the development of diet induced obesity [13, 14]. Furthermore, PC, CA, and K-*O*-R have been reported to have anti-inflammatory [15], antioxidant [16], and anti-hepatitis B virus activity [17] and have been shown to inhibit hepatocarcinogenesis [18] and have analgesic activity [19].

Recently, we reported that SV extracts and isolates of PC, CA, and K-*O*-R have a strong antiadipogenic effect in 3T3-L1 cells through the suppression of increases in PPAR-*γ* and C/EBP*α* expression [20] and decrease body and fat tissue weight, as well as lower low-density lipoprotein-cholesterol and triglycerides levels in the blood [21]. Therefore, these three compounds were considered to be the best bioactive markers for standardized extracts of SV for use for antiobesity functional food ingredients.

In the present study, we collected various SV plants in Korea including the Ulsan, Goseong, Daegwallyeong, Geoje, Halla Mountain, Ulleung Island, and Wi Island. Extracts of these were assessed for antiadipogenesis activity in 3T3-L1, as well as SV plant extracts taken in different time periods (April, May, June, July, and August). We determined the amount of the three active components using a validated simultaneous high performance liquid chromatography (HPLC) method and show a correlation between antiobesity activity and the quantity of PC, CA, and K-*O*-R. From this, we determined the most suitable time periods and regions for efficient standardization of SV extracts for use as an antiobesity functional ingredient.

## 2. Materials and Methods

### 2.1. Chemicals and Reagents

Deionized water was purified using a Milli-Q laboratory water purification system (Millipore, Bedford, MA, USA). All reagents and solvents were purchased from Sigma-Aldrich Co. (St. Louis, MO, USA). PC, CA, and K-*O*-R were isolated and purified as previously described [20].

### 2.2. Collection and Extraction of* Solidago virgaurea* subsp.* gigantea*

SV plants of different regions were provided and harvested from either USE, GSE, DGLE, GJE, HLME, ULIE, or WIE from the Kugya Farm in Chuncheon, South Korea (Oct, 2014). SV plants of different periods were collected in April, May, June, July, and August from the Agriculture Technology Center in the Ulleung Island, South Korea (2014). In addition, extracts were collected from ULIE in April, May, June, July, and August and were named APR-E, MAY-E, JUN-E, JUL-E, and AUG-E, respectively. The dried SVs (1.5 kg) were crushed and then were extracted with 10% ethanol (EtOH, 15 L) at 70°C for 7 h. The extracts were then concentrated by reduced pressure evaporator (N-1000, Tokyo Rikakikai, Tokyo, Japan) and finally were freeze-dried using PVTFD10R (Ilshinbiobase Co., Ltd., Yangju, Korea) to obtain a solid powder.

### 2.3. 3T3-L1 Cell Culture and Adipocyte Differentiation

3T3-L1 fibroblasts were obtained from the American Type Culture Collection (Manassas, VA, USA) and grown to confluency at 37°C under a humidified 5% CO_2_ atmosphere in Dulbecco's Modified Eagle's Medium (DEME, Gibco, Waltham, MA, USA), containing 10% bovine calf serum (GenDEPOT, Katy, TX, USA), and 100 U/ml penicillin-streptomycin (Gibco). Two days after the cells had reached confluency (day 0), preadipocytes of 3T3-L1 were cultured in differentiation medium (DM) containing 10% fetal bovine serum (FBS, Gibco), 10 *μ*g/ml insulin (Sigma-Aldrich), 0.5 mM 3-isobutyl-1-methylxanthine (IBMX, Sigma-Aldrich), and 1 *μ*M dexamethasone (Sigma-Aldrich). Two days after stimulation with differentiation inducer (MDI, including 0.5 mM IBMX, 1 *μ*M dexamethasone, and 10 *μ*g/ml insulin) (day 2), the medium was switched to one containing 10% FBS and 10 *μ*g/ml insulin. Two days later (day 4), the medium was changed to 10% FBS/DMEM. The cells were cultured in 10% FBS/DMEM every 2 days. Full differentiation was achieved by day 8. The extract samples were added to the 3T3-L1 culture at the concentration of 10 and 50 *μ*g/ml on day 4 after differentiation induction [22].

### 2.4. Oil Red O Staining

To determine adipogenic potential and fat accumulation, we stained the cells with Oil Red O solution (Sigma Chemical Co., St. Louis, MO). On day 8, the cultured 3T3-L1 cells were washed with phosphate buffered saline (PBS) and then fixed with 10% formaldehyde at room temperature. The cells were stained with 0.5 *μ*g/ml Oil Red O solution. After the Oil Red O staining, cells were photographed using an optical microscope system (Axiomager, Zeiss, Germany) at 100x magnification. The lipid droplets were dissolved in isopropanol and absorbance was measured at 540 nm using a microplate reader (Sensident scan, Labsystems, Helsinki, Finland). The relative lipid content and percent adipogenesis inhibition was calculated using the following equations: Relative lipid content (%) = (Sample  OD/Control  OD) × 100; Inhibition  (%) = (1 − [sample  OD − control  OD]/[DM  OD − control  OD]) × 100.

### 2.5. MTS Assay

The cytotoxicity of the SV extracts on 3T3-L1 cells was examined using [3-(4,5-dimethylthiazol-2-yl)-5-(3-carboxymethoxyphenyl)-2-(4-sulfophenyl)-2H-tetrazolium, inner salt] (MTS) assay kit (Promega, Madison, WI, USA) [23]. Cells (5 × 10^3^/well) were cultured in 96-well plates and treated with the SV extracts (10 and 50 *μ*g/ml) for 24 h. After incubation, 20 *μ*l/well of MTS solution was incubated for 20 min at 37°C in a humidified 5% CO_2_ atmosphere. Optical density at 490 nm was measured three times using a microplate reader (Sensident scan).

### 2.6. Preparation of Standards and Analytical Samples

Standard solutions of the PC, CA, and K-*O*-R were prepared at concentrations of 4.85−485.00, 47.50−1900.00, and 8.5−850.00 *μ*g/ml, respectively, by dissolving the samples in MeOH. SV extracts were prepared by weighing 10.0 mg of the sample in volume flask and dissolving with 1.0 ml MeOH. Each sample was sonicated for 10 min to ensure complete dissolution. Standard three solutions were analyzed in triplicate and filtered through a 0.45 *μ*m nylon membrane filter prior to analysis. All analytical solutions were stored at 0°C before use.

### 2.7. HPLC and Chromatographic Analytical Conditions

The SV extracts were analyzed using an Agilent 1200 liquid chromatographic system (Agilent Technologies, Santa Clara, CA, USA) with an Eclipse XDB-C18 column (150 × 4.6 mm, 5 *μ*m, Agilent). The mobile phase consisted of A (0.1% trifluoroacetic acid) and B (MeOH); the gradient was as follows: 5−40% B (0−15 minutes), 40−60% B (15−30 minutes), and 60−100% B (30−40 minutes) at a flow rate of 0.7 ml/min. The UV diode array detector was set at 254 nm and sample injection volume was 10 *μ*l at a column temperature of 30°C.

### 2.8. Analytical Method Validation

The analytical method was validated according to the Guidelines for Single Laboratory Validation of Chemical Methods for Dietary Supplements and Botanicals of AOAC [24]. The validation parameters included specificity, linearity, LOD, LOQ, precision, accuracy, range, and recovery.

#### 2.8.1. LOD and LOQ

The linearity of sample concentration was evaluated across the range of 4.85–485.00 *μ*g/ml for PC, 47.5−1900.00 *μ*g/ml for CA, and 8.50−850.00 *μ*g/ml for K-*O*-R. Standard solutions of the three bioactive compounds were diluted with MeOH to five concentrations appropriate for plotting the calibration curves. The different concentrations of each analyte were injected in triplicate. To substantiate the linearity of the analytical method validation, calibration curves were constructed from the peak area versus the concentration of the standards. The SD of the response and the slopes of the concentration curves of the calibration curves were used to estimate the LOD and LOQ. The LOD and LOQ were calculated using the following equations: LOD = 3.3*σ*/*S*; LOQ = 10*σ*/*S*; *σ* is the residual SD of the regression line; and *S* is the slope of the standard curve.

#### 2.8.2. Precision

The retention times of each standard in the SV extract were identified and the percentage of relative standard deviation (% RSD) was calculated to confirm the specificity of the peaks. Evaluation of method repeatability (intraday precision) and reproducibility (interday precision) was performed. Standard solutions at three different concentrations were analyzed. Intraday precision was determined from three replications within 1 day, and the interday precision was analyzed in three replications in different days, conducted over 3 days. The precision of the method was expressed as the % RSD for each test; a value of RSD within 3% is generally considered acceptable.

#### 2.8.3. Accuracy and Recovery

Accuracy was evaluated across the specified range of the analytical procedure by a recovery study. Preanalyzed standard solutions were used for comparison. Three different concentrations of standards were spiked into the sample extract in triplicate. The percentage of recovery of each compound was analyzed using the validated method. Recovery was estimated using the following formulae: recovery (%) = ([recovered  amount − original  amount/spiked  amount]) × 100.

### 2.9. Statistical Analysis

Data are expressed as mean value ± SE and comparisons of data were carried out using Student's unpaired* t*-test or one-way ANOVA, as appropriate. *P* < 0.05 was considered statistically significant.

## 3. Results and Discussion

### 3.1. Validation of the Analytical Method

#### 3.1.1. Optimization of the HPLC Analytical Conditions

**Figure 1:**
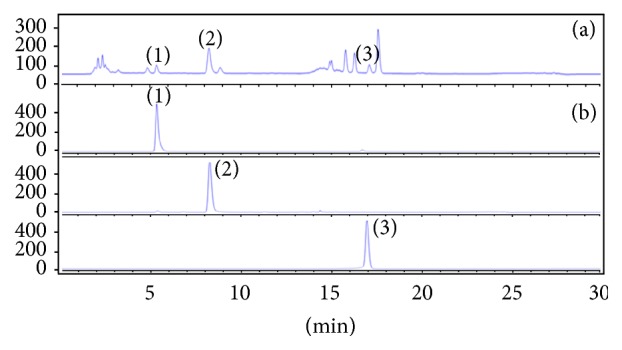
HPLC chromatogram of SV extract collected in Ulleung Island in April at 254 nm detection. Peaks of SV aqueous extract (a) and standardization of the three marker compounds (b): (1) protocatechuic acid; (2) chlorogenic acid; (3) kaempferol-3-*O*-rutinoside.

For the analysis, we optimized the HPLC conditions to obtain high separation and resolution of PA, CA, and K-*O*-R. To enhance chromatographic separation and resolution capacity, 0.1% TFA (v/v) water and MeOH were used as mobile solvents with a gradient elution system. The detector was set to 254 nm. The chromatogram of the standards of the three compounds is shown in Figure 1. Good separation could be achieved within 20 min. The retention time for three compounds was 5.36, 8.22, and 17.04 min, for PA, CA, and K-*O*-R, respectively. These results indicate that these HPLC analytical conditions result in appropriate selectivity and specificity.

#### 3.1.2. Linearity, Limit of Detection (LOD), and Limit of Quantification (LOQ)

Linearity, LODs, and LOQs were examined for the validated method. Calibration curves were determined from linear ranges of concentrations (4.85−485.00 *μ*g/ml for PA, 47.50−1900.00 *μ*g/ml for CA, and 8.50−850.00 *μ*g/ml for K-*O*-R). All calibration curves showed good linearity (*r*^2^ > 0.999) within their respective concentration ranges. The LOD of PA, CA, and K-*O*-R was found to be 0.208, 3.088, and 1.439 *μ*g/ml, respectively, and the LOQ of these compounds was found to be 0.630, 9.358, and 4.360 *μ*g/ml, respectively (Table 1).

#### 3.1.3. Precision

The precision of the method was evaluated by assessing the relative standard deviation (RSD) intraday and interday at three different concentrations. Intraday and interday tests were performed by applying three different concentrations of standard, in triplicate, three times a day on three different days. The RSDs (%) for PA, CA, and K-*O*-R were less than 2% (Table 2).

#### 3.1.4. Accuracy and Recovery

The accuracy of the validated method was calculated by using a spiking technique. As shown in Table 3, the recoveries of PA, CA, and K-*O*-R ranged from 101.32 to 103.30%, 95.82 to 100.25%, and 96.18 to 99.37%, respectively.

### 3.2. Effect of Harvesting Location of SV on Preadipocyte Viability

**Figure 2:**
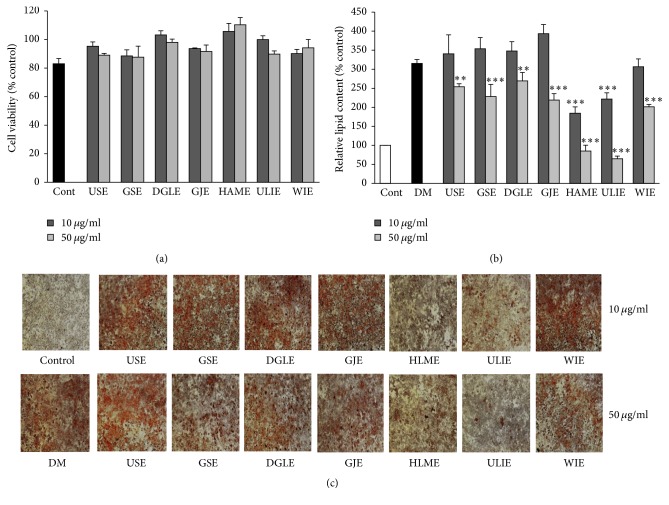
Antiadipogenesis effect on 3T3-L1 of SV extracts collected from different regions. (a) Effect of SV extracts at 10 and 50 *μ*g/ml on viabilities of 3T3-L1 cells determined by MTS assay for 24 h; (b) relative lipid content, quantified by absorbance in 3T3-L1 cells with or without SV extracts at 10 and 50 *μ*g/mL for 8 days; (c) Oil Red O staining of lipid droplets in 3T3-L1 cells with or without SV extracts at 10 and 50 *μ*g/mL for 8 days. Results are presented mean ± SE. The asterisk indicates a significant difference compared to DM (^*∗∗*^*P* < 0.01 and ^*∗∗∗*^*P* < 0.001).

Cell viability of SV extracts from SV of different regions was evaluated at 10 and 50 *μ*g/ml on 3T3-L1 cells. As shown in Figure 2(a), the SV extracts at all concentrations had no significant effects of viability after 24 h of treatment at these concentrations. Also, as shown in Figures 2(b) and 2(c), treatment of the extract with SV from Halla Mountain (HLME) and Ulleung Island (ULIE) significantly decreased lipid accumulation of 3T3-L1 adipocyte cells at concentrations 10 and 50 *μ*g/ml. The percentage of lipid content decreased from 43.37% to 106.89%, with the highest declines in the cells being those treated with HLME and the ULIE SV extract.

### 3.3. Effect of Harvesting Time on Preadipocyte Viability and Adipocyte Differentiation in 3T3-L1 Cells of ULIE SV Extract

**Figure 3:**
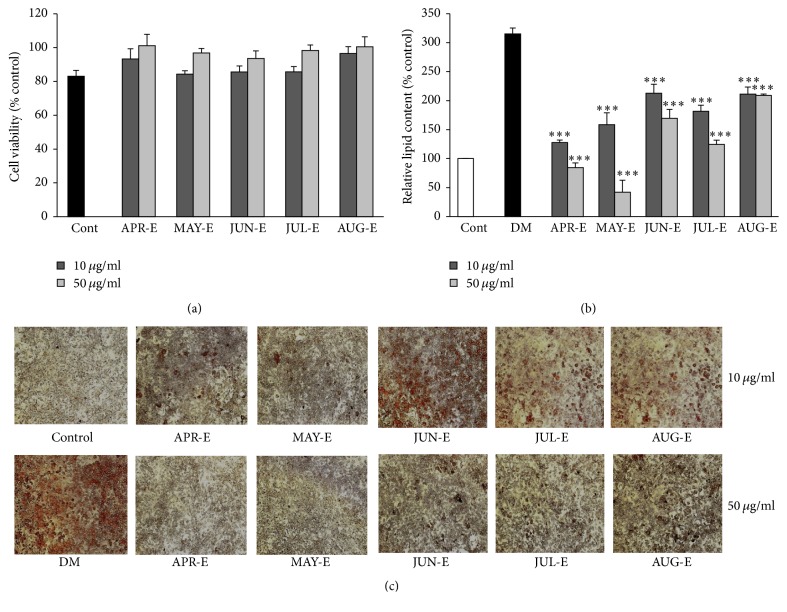
Antiadipogenesis effect on 3T3-L1of SV extracts from different harvesting times from Ulleung Island. (a) Effect of SV extracts on 3T3-L1 cell viability determined by MTS assay at 10 and 50 *μ*g/ml for 24 h; (b) relative lipid content quantified by absorbance in 3T3-L1 cells with or without SV extract at 10 and 50 *μ*g/mL for 8 days; (c) Oil Red O staining of lipid droplets in 3T3-L1 cells treatment with or without SV extract at 10 and 50 *μ*g/mL for 8 days. Results are presented mean ± SE. The asterisk indicates a significant difference compared to DM (^*∗∗∗*^*P* < 0.001).

The extract with SV from ULIE significantly decreased lipid accumulation. Therefore, we used this region to investigate the cytotoxicity of SV extracts from SV that was collected at different times. As shown in Figure 3(a), the SV extracts at 10 and 50 *μ*g/ml had no statistically significant growth inhibition effects on cell viability of preadipocyte using the MTS assay after 24 h treatment. The effects of SV extracts from different periods on antiadipogenesis in 3T3-L1 cells were analyzed quantitatively with Oil Red O staining at a concentration of 10 and 50 *μ*g/ml, at the differentiation stage of the cell. As shown in Figures 3(b) and 3(c), Oil Red O staining of the cells revealed that accumulation of lipid droplets in 3T3-L1 adipocytes decreased significantly following treatment with SV extracts from plants harvested at different times at 10 and 50 *μ*g/ml concentrations. The extract from SV harvested in April was observed to have the highest inhibitory effects of 87.14 and 110.70% on adipogenesis at 10 and 50 *μ*g/ml concentrations.

### 3.4. Quantification of PA, CA, and K-*O*-R in SV Extracts Based on HPLC Determination

The validated HPLC method was used to measure the quantity of PA, CA, and K-*O*-R in the SV extracts collected from different regions and periods. The results are shown in Tables 4 and 5. The amount of PA, CA, and K-*O*-R in the SV extracts from SV harvested at different time periods ranged from 1.09 to 4.38, 0.42 to 35.54, and 3.47 to 4.43 mg/g, respectively (Table 4). The highest content of PA, CA, and K-*O*-R in SV extracts from ULIE at different times was found in the samples harvested in May, April, and April, respectively. The quantity of PA, CA, and K-*O*-R in the SV extracts from different regions was in the ranges of 0.97–4.7, 3.28–78.71, and 81.47–776.90 mg/g, respectively (Table 5). The highest PA, CA, and K-*O*-R content was found in the extracts of SV from Daegwallyeong (DGLE), HLME, and HLME, respectively.

Obesity leads to a variety of metabolic diseases such as hypertension, hyperlipidemia, cardiovascular disease, diabetes, cancers, and nonalcoholic fatty liver disease [24]. As adipocytes are the main site for adipogenesis and accumulation of triglycerides, they are a useful focus for studies on antiobesity. The 3T3-L1 cell line is widely used to develop antiadipogenesis agents through adipocyte differentiation [25]. Our previous studies showed that SV extracts decrease body weight, fat tissue weight, and low-density lipoprotein-cholesterol and triglycerides levels in blood [26]. In addition, the* p*-AMP-activated protein kinase (AMPK) level in the fat tissue of SV-extract-treated SD rats increased. The levels of AMPK-downstream proteins, such as the* c*-AMP response element binding protein and acetyl-CoA carboxylase, fatty acid synthase, and FABP4, all decreased, indicating SV-extract-activated AMPK inhibited adipogenesis and lipid biosynthesis in fat tissue [21]. PA, CA, and K-*O*-R isolated from SV extract inhibited adipocyte differentiation in 3T3-L1 cells. To date, many studies on the effect of PA, CA, and K-*O*-R have shown that PA, CA, and K-*O*-R improve glucose tolerance and insulin sensitivity in obese mice, increase lipid metabolism, and have antiadipogenic activity [12]. In particular, K-*O*-R has shown antiadipogenic activity in 3T3-L1 cells by downregulating the expression of PPAR-*γ* and C/EBP-*α* [20]. PA in* Rubus coreanus* inhibited lipid accumulation in adipocytes [27]. Koo et al. (2014) suggested that PA significantly reduced the total cholesterol, TG, and LDL-c level and increased the HDL-c level and reduced the levels of GOT and GPT in high-cholesterol-diet induced mice [28]. In addition, CA decreased fasting plasma glucose, glycosylated hemoglobin, and visceral fat content levels in db/db diabetic mice and improved lipid metabolism through PPAR-*α* [29]. CA improved blood lipid metabolism in rats by alleviating the levels of free fatty acid and triglycerides in liver through AMPK pathway [30]. Therefore, the PA, CA, and K-*O*-R isolated from SV extract not only have enhanced antiadipogenic activity but also could have antiobesity activity in humans.

To analyze the association between antiadipogenesis activity and the presence of the three bioactive compounds in SV extracts of different periods and regions, we developed a quick and efficient method of analysis using HPLC. The HPLC method was validated by optimizing linearity, LOD, LOQ, precision, and accuracy. The resulting HPLC method showed excellent linearity, LOD, LOQ, precision, and accuracy, with a RSD < 2%. Relatively high contents of PA, CA, and K-*O*-R were observed from the DGLE and HLME samples. The following order of average K-*O*-R content was observed: HLME (776.90 g/mg) > ULIE (310.62 mg/g) > Goseong (GSE) (103.20 mg/g) > Wi Island (WIE) (81.47 mg/g); K-*O*-R was not observed in the extracts of SV from Ulsan (USE), DGLE, or Geoje (GJE). CA content was found in the following order: HLME (78.71 mg/g) > WIE (36.24 mg/g) > GSE (33.62 mg/g) > ULIE (81.47 mg/g) > USE (776.90 mg/g) > DGLE (310.62 mg/g) > GJE (103.20 mg/g). PA content was found in the following order: DGLE (4.70 mg/g) > WIE (3.37 mg/g) > ULIE (3.17 mg/g) > HLME (1.99 mg/g) > USE (1.46 mg/g) > GJE (1.04 mg/g) > GSE (0.97 mg/g). Our results show that our method is a useful tool for the standardization of SV extracts. Previously, Chandra and Sharma reported a rapid quantitative determination of caffeine and paracetamol in formulated tablets by reverse-phase HPLC and gradient elution using MeOH-water as mobile phase at 40 : 60 (v/v) with a flow rate of 1.0 min/mL and detection at 243 nm [31]. A simple, sensitive, and precise reversed-phase liquid chromatographic method was developed for the quantitative determination of four phenolic compounds (gallic acid, fustin, fisetin, and sulfuretin) from the stem extract of* Rhus verniciflua* stokes by Kim et al. (2013) [32]. Savic et al. (2013) developed and validated an reversed-phase HPLC method for determination of quercetin in green tea that is simpler and faster than other available methods, with a flow rate of 1.0 mL/min, a C18 column (4.6 × 250 mm, 5 *μ*m), and detection at 370 nm [33]. These researchers suggested that the HPLC method that was developed could be successfully used for the quantification of compounds in natural extracts and foods. Based on these results, we also suggest that HPLC can provide a quantitative basis for quality control of SV extracts.

As shown in Tables 4 and 5, the quantity of PA in SV extracts is similar in extracts from the five time periods and seven regions, while the ULIE and HLME samples contained the highest quantity of CA and K-*O*-R from different periods and regions. Moreover, we ascertained that the highest antiadipogenesis activity of SV extracts in 3T3-L1 cells was higher in ULIE than HLME. However, the SV extract of five different time periods showed different amounts of CA and K-*O*-R in the ULIE samples. Of those taken at different time periods, the highest CA levels were found in the APR-E sample; the K-*O*-R content was lower in ULIE. Likewise, K-*O*-R content was highest in HLME sample; however, HLME showed lower antiadipogenesis activity than the ULIE samples. There was no significant relationship between the amount of the three compounds and the antiadipogenesis effects of the SV extracts from different time periods and regions. Our study suggests that (1) an antiadipogenesis effect is induced by high concentrations of the CA and K-*O*-R in SV extracts; (2) there is a synergistic effect between CA and K-*O*-R; and (3) CA and K-*O*-R could be useful active ingredients for antiadipogenesis, and a proprietary ratio of CA and K-*O*-R in an SV extract could be a functional food resource. Based on these results from different time periods and SV extract regions, we suggest that samples collected in April from the Ulleung Island can be used to make functional food ingredients preventing obesity.

## 4. Conclusion

In summary, to standardize the SV extract as a functional food ingredient, we established and validated a novel HPLC method for the simultaneous determination of three bioactive compounds in SV extracts of different times and regions of harvest. Our study successfully confirmed different antiobesity effects and quantities of three bioactivity compounds, without cytotoxicity in 3T3-L1 cells, of SV extracts harvested in different times and from different regions, using HPLC. SV extracts collected in April from Ulleung Island show more promise as functional food ingredients preventing obesity, as the PA, CA, and K-*O*-R content was 2.77, 35.54, and 4.43 mg/g, respectively. Therefore, Ulleung Island from April exerts antiobesity effects by suppressing adipogenesis and can be considered a useful functional food resource for preventing obesity. In addition, our HPLC method was accurate and reproducible and can provide a quantitative basis for quality control of SV extracts.

## Figures and Tables

**Table 1:** Method validation parameters for quantitation of protocatechuic acid, chlorogenic acid, and kaempferol-3-*O*-rutinoside.

Parameters	Protocatechuic acid (PC)	Chlorogenic acid (CA)	Kaempferol-3-*O*-rutinoside (K-*O*-R)
Retention time (min; *n* = 4)	5.36 ± 0.002	8.22 ± 0.004	17.04 ± 0.002
Regression equation^a^	*Y* = 28.899*x* − 125.76	*Y* = 12.405*x* + 33.466	*Y* = 22.481*x* + 196.82
Correlation coefficient (*R*^2^)	1.0000	0.9999	0.9989
Linear range (*μ*g/ml)	4.85–485.00	47.5–1900.00	8.50–850.00
LOQ (*μ*g/ml)^b^	0.630	9.358	4.360
LOD (*μ*g/ml)^c^	0.208	3.088	1.439

^a^ *X*: concentration of tested compounds (1)–(3); *Y*: peak area at 254 nm; ^b^LOQ: 10 × (standard deviation of the response/slope of calibration curve); ^c^LOD: 3.3 × (standard deviation of the response/slope of calibration curve).

**Table 2:** Precision (intraday and interday) and accuracy of protocatechuic acid, chlorogenic acid, and kaempferol-3-*O*-rutinoside detection.

Analyte compounds	Analyte concentration (*μ*g/ml)	Intraday (*n* = 3)			Interday (*n* = 3)		
		Calculated concentration (mean ± SD, *μ*g/ml)	RSD (%)	Accuracy (%)	Calculated concentration (mean ± SD, *μ*g/ml)	RSD (%)	Accuracy (%)
Protocatechuic acid (PC)	18.92	19.02 ± 0.01	0.07	99.48	19.02 ± 0.04	0.23	99.46
	23.77	24.21 ± 0.32	1.33	98.19	23.90 ± 0.05	0.21	99.46
	62.57	64.25 ± 0.08	0.12	97.38	64.17 ± 0.08	0.12	97.50

Chlorogenic acid (CA)	253.07	249.58 ± 1.45	0.58	101.4	251.08 ± 0.03	0.01	100.79
	300.57	249.58 ± 1.59	0.53	100.46	300.81 ± 0.37	0.12	99.92
	680.57	661.17 ± 1.62	0.24	102.93	662.99 ± 0.65	0.10	102.65

Kaempferol-3-*O*-rutinoside (K-*O*-R)	24.45	24.49 ± 0.14	0.57	99.81	24.46 ± 0.06	0.25	99.93
	58.45	56.06 ± 0.18	0.32	104.26	56.67 ± 0.19	0.34	103.14
	100.95	97.70 ± 0.23	0.24	103.33	96.61 ± 0.68	0.70	104.49

**Table 3:** Recovery of protocatechuic acid, chlorogenic acid, and kaempferol-3-*O*-rutinoside.

Analyte compounds	Theoretical (*μ*g/ml)	Found (mean ± SD, *μ*g/ml)	RSD (%)	Recovery (mean ± SD, %)
Protocatechuic acid (PC)	4.85	4.95 ± 0.87	0.87	102.12 ± 0.00
	9.70	9.83 ± 0.05	0.21	101.32 ± 0.51
	48.50	50.10 ± 0.08	0.12	103.30 ± 0.16

Chlorogenic acid (CA)	47.50	45.51 ± 0.03	0.07	95.82 ± 0.07
	95.00	95.24 ± 0.37	0.39	100.25 ± 0.39
	475.00	457.63 ± 0.65	0.14	96.30 ± 0.14

Kaempferol-3-*O*-rutinoside (K-*O*-R)	8.5	8.45 ± 0.03	0.34	99.37 ± 0.34
	42.5	40.93 ± 0.07	0.18	96.31 ± 0.17
	85	81.75 ± 0.23	0.29	96.18 ± 0.28

**Table 4:** Quantification of protocatechuic acid, chlorogenic acid, and kaempferol-3-*O*-rutinoside from *Solidago virgaurea *subsp. *gigantea* sample collection at different times.

Periods (month)	Contents in extracts (mean ± SD, mg/g)		
	Protocatechuic acid (PC)	Chlorogenic acid (CA)	Kaempferol-3-*O*-rutinoside (K-*O*-R)
April (APR-E)	2.77 ± 0.04	35.54 ± 0.03	4.43 ± 0.02
May (MAY-E)	4.38 ± 0.01	23.45 ± 0.00	—
June (JUN-E)	3.80 ± 0.00	25.82 ± 0.04	—
July (JUL-E)	1.09 ± 0.01	15.49 ± 0.04	3.47 ± 0.01
August (AUG-E)	—	0.42 ± 0.01	—

**Table 5:** Quantification of protocatechuic acid, chlorogenic acid, and kaempferol-3-*O*-rutinoside from *Solidago virgaurea *subsp. *gigantea* samples from different regions.

Regions	Contents in extracts (mean ± SD, mg/g)		
	Protocatechuic acid (PC)	Chlorogenic acid (CA)	Kaempferol-3-*O*-rutinoside (K-*O*-R)
Ulsan (USE)	1.46 ± 0.00	12.43 ± 0.06	—
Goseong (GSE)	0.97 ± 0.00	33.62 ± 0.08	103.20 ± 0.71
Daegwallyeong (DGLE)	4.70 ± 0.00	7.28 ± 0.04	—
Geoje (GJE)	1.04 ± 0.80	3.28 ± 0.01	—
Halla Mountain (HLME)	1.99 ± 0.04	78.71 ± 0.26	776.90 ± 0.26
Ulleung Island (ULSE)	3.17 ± 0.03	26.25 ± 0.01	310.62 ± 0.51
Wi Island (WIE)	3.37 ± 0.00	36.24 ± 0.12	81.47 ± 1.32
